# Downscaling MERRA-2 Reanalysis PM_2.5_ Series over the Arabian Gulf by Inverse Distance Weighting, Bicubic Spline Smoothing, and Spatio-Temporal Kriging

**DOI:** 10.3390/toxics12030177

**Published:** 2024-02-25

**Authors:** Youssef Saliba, Alina Bărbulescu

**Affiliations:** 1 Doctoral School, Technical University of Civil Engineering of Bucharest, 122-124 Lacul Tei Av., 020396 Bucharest, Romania; youssefsaliba@gmail.com; 2 Department of Civil Engineering, Transilvania University of Brașov, 5 Turnului Str., 900152 Brașov, Romania

**Keywords:** PM_2.5_, spatial interpolation, IDW, BS, STK

## Abstract

This study offers a detailed analysis of the fine particulate matter (PM_2.5_) series in the Arabian Gulf zone, employing three interpolation models, Inverse Distance Weighting (IDW), Bicubic Spline Smoothing (BSS) and Spatio-Temporal Kriging (STK). Unique advancements include the use of complete temporal records in IDW, the management of edge effects in S with synthetic buffer points, and the application of STK to detrended data residuals. The results indicated that the BBS, particularly adept at handling boundary conditions, significantly outperformed the other methods. Compared to IDW, the Mean Absolute Error (MAE), Root Mean Square Error (RMSE), and Mean Absolute Percentage Error (MAPE) decreased by 21%, 15%, and 21%, respectively, in BSS. Compared to STK, MAE, RMSE, and MAPE were lower with around 60%, 61%, and 58%, respectively in BSS. These findings underscore the efficacy of the BSS method in spatial interpolation for environmental monitoring, contributing to enhanced PM_2.5_ analysis and public health management in the region.

## 1. Introduction

Fine particulate matter (PM_2.5_) is a mixture of solid particles and liquid droplets with diameters lower than 2.5 μm, easily inhalable, and found in the air. They contain metallic and organic compounds, elemental carbon, inorganic ions, etc. [1]. Particulate matter (PM) mainly originates from fossil fuel or wood combustion, volcanoes, forest fires, the Earth’s crust degradation, dust storms, sea spray, and biological emissions [2,3,4]. They may also result from chemical reactions between precursor gases [5]. Due to their size, density, and atmospheric conditions, PM_2.5_ remains suspended, polluting the air [6]. They can be inhaled and deposited on the lung surface, damaging tissues and producing inflammation. The most affected are the children and adults who already suffer from lung or heart diseases.

Exposure for up to 24 h leads to respiratory symptoms, asthma attacks, and acute and chronic bronchitis, whereas exposure for months or years results in premature death [7]. The report on the ambient air quality indicates that in California, during 2014–2016, 2800 hospitalizations/year for respiratory and cardiovascular diseases and 6700 visits/year to emergency rooms due to asthma provoked or aggravated by PM_2.5_ were noticed [1]. Statistics show that PM_2.5_ is responsible for about 4 million deaths from respiratory infections, chronic lung disease, cancers, heart disease, preterm births, etc. A review of the harmful effects of PM_2.5_ on the organism is presented in [8]. 

The World Bank statistics indicate that in the United Arab Emirates (UAE), the mean annual exposure to PM_2.5_ surpasses eight times the safety limits established by the World Health Organization (WHO). Even if the sandstorms contribute to the deterioration of the air quality, studies indicate that the emissions (mainly from fossil fuels) represent the primary source of air pollution in the UAE [9,10,11,12,13]. In September 2023, the 30 governmental monitoring stations in the UAE indicated that the PM_2.5_ levels were three times higher than the warning limits established in 2021 by WHO. 

One-fifth of the UAE’s anthropic pollution with PM_2.5_ is due to road transport, whereas about two-thirds result from industry [14]. In 2019, the mean annual exposure to PM_2.5_ in the UAE was 44 μg/m^3^, compared to the global average—46, USA—8, Brazil—13, Russia—12, China—48, Saudi Arabia—62, India—83, and Qatar—76 [15,16]. 

Given the impacts on human health and public health management, many studies focus on monitoring and estimating pollutant concentration [17,18]. Due to the limited number of monitoring stations, spatial interpolation is necessary to evaluate PM concentrations at places where data are not available. Still, one must consider that PM_2.5_ can vary rapidly from one site to the other, and a minimum number of stations is necessary to produce a good mapping. Various methods have been used for this goal. They include Inverse Distance Weighting (IDW) or some of its versions for PM_2.5_ trend evaluation [19,20], kriging [21,22], neural networks [23,24,25,26], generalized additive mixed models [27], Multiscale Geographically Weighted Regression (MGWR) [28], Bayesian Kriging, and Tensor Spline Function [29], Random-forest-spatio-temporal kriging [30], hierarchical modeling [31], hybrid models [32], data fusion [33], optimal interpolation [34], exponentially smoothing [35], etc. Each one addressed some issues of the classical interpolation techniques, intending to increase the forecast accuracy. 

This article’s aim is in range with the mentioned approaches. It fills a gap in the knowledge of the PM_2.5_ series analysis carried out by other scientists (using statistical analysis [36] and PM_10_/PM_2.5_ ratio [37]) by introducing a refined version of Bicubic Spline Smoothing (BSS). It enhances the estimation of PM_2.5_ concentrations by effectively managing corner and edge effects, integrating synthetic buffer points around the spatial domain of the data with values derived from the mean of adjacent real data points, thereby ensuring the continuity and precision of the study area’s periphery. Complementing this, we also present an adapted IDW technique specifically tailored for spatio-temporal analysis. Incorporating the complete temporal records from each monitoring station into the Leave-one-Out-Cross-validation (LOOCV) process and optimizing the power parameter based on spatial and temporal data. Furthermore, we explore a spatio-temporal kriging (STK) approach, underlining the critical role of detrending in spatio-temporal analysis. The STK technique first employs a Generalized Additive Mixed Model (GAMM) to isolate the residuals for subsequent analysis. It then selects the best fit from various variogram models using a Bayesian approach. This dual-phase process, combining GAMM detrending with STK, allows us to capture the nuanced spatio-temporal dynamics of PM_2.5_ concentrations, ensuring a robust interpolation that accounts for both spatial correlations and temporal variability.

Given the high pollution recorded, the Arabian Gulf zone was chosen for this study. Despite some reports providing data on high PM_2.5_ concentrations in the UAE, no extended study was performed. Moreover, reliable interpolation methods can provide values of the PM_2.5_ concentrations at places where measurements are not available. The results of the study could be used by the decision factors for taking insight measures for pollution reduction (and, implicitly, reducing its impact on population health).

## 2. Materials and Methods

### 2.1. Data Series

The second Modern-Era Retrospective analysis for Research and Applications (MERRA-2) is produced by the NASA Global Modeling and Assimilation Office (GMAO) [38,39,40] using the Goddard Earth Observing System, version 5 (GEOS-5) and data assimilation system [41]. The GEOS-5 incorporates atmospheric composition and circulation, land and oceanic components, as well as aerosol processes based on the Goddard Chemistry, Aerosol, Radiation, and Transport (GOCART) model, and can analyze the atmosphere composition and interactions between the climate and aerosols [41,42,43].

Apart from meteorological parameter data assimilation [44], MERRA-2 incorporates bias-corrected 550 nm aerosol optical depth (AOD) from assimilation from Advanced Very High-Resolution Radiometer, Moderate Resolution Imaging Spectroradiometer, assimilated the non-bias-corrected 550 nm aerosol optical depth from the Multiangle Imaging Spectroradiometer, and ground-sources (Aerosol Robotic Network stations) [37,40,44,45].

Based on GEOS-5 and GOCART, CMAO simulated the following aerosol types: sulfate (SO_4_), black and organic carbon (BC and OC, respectively), sea salt (SS), and dust (Dust). The PM_2.5_ concentrations were computed in MERRA-2 using the concentrations from the GOCART aerosol module, with

(1) $${PM}_{2.5}=1.375\times {SO}_{4}+BC+1.6\times OC+{SS}_{2.5}+{Dust}_{2.5},$$

where the index 2.5 at SS and Dust indicates that the PM diameter is less than 2.5 µm [46].

Information on PM_2.5_-related chemical composition is provided by satellite-derived products. Optical signals fall in this class, which must be modeled for obtaining surface mass concentrations. Due to clouds and high surface albedo, missing data (nonrandom) are inherent, raising difficulties in quickly obtaining updated PM_2.5_ components’ concentrations with full spatial coverage. The reanalysis data provide the optimal estimates of these concentrations. Variational data assimilation algorithms can produce error estimates for the studied variables. Although they are concordant with the background and observation error statistics, they are challenging to obtain and limited by the algorithm’s assumed errors. So, the main uncertainties are due to the algorithms used for modeling the signals to obtain surface mass concentration, ‘completing’ the randomly missing data, and the errors from the data assimilation algorithms [47,48,49,50,51,52].

In this research, we used the Dust Column Mass Density PM_2.5_ concentration (μg/m^3^) series for January 1990–April 2017 from the tavgM_2d_aer_Nx, which is a time-averaged two-dimensional monthly mean data collection in MERRA-2 [53]. 

**Figure 1:**
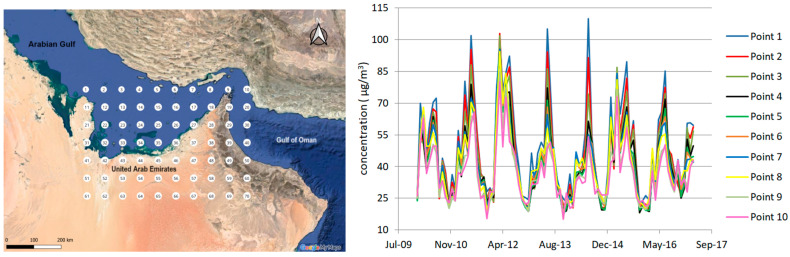
(**left**) Map of the region and the positions of the observation points; (**right**) the data series recorded at the first ten points.

The MERRA-2 grid points are represented on the map in Figure 1 (left), and their coordinates are given in Table A1. They are in the Arabian Gulf zone and over the United Arab Emirates. The dataset is complete (without gaps). The series from the first ten points are presented in Figure 1 (right). All exhibit a similar seasonal trend.

The range of the basic statistics of the data series is presented in Table 1. There are significant differences between the series values, indicated, for example, by the range of the average values—[3.65 × 10^−8^, 1.19 × 10^−7^], maximum values—[8.01 × 10^−8^, 2.46 × 10^−7^], and standard deviations—[1.31 × 10^−8^, 3.71 × 10^−8^]. 

**Figure 2:**
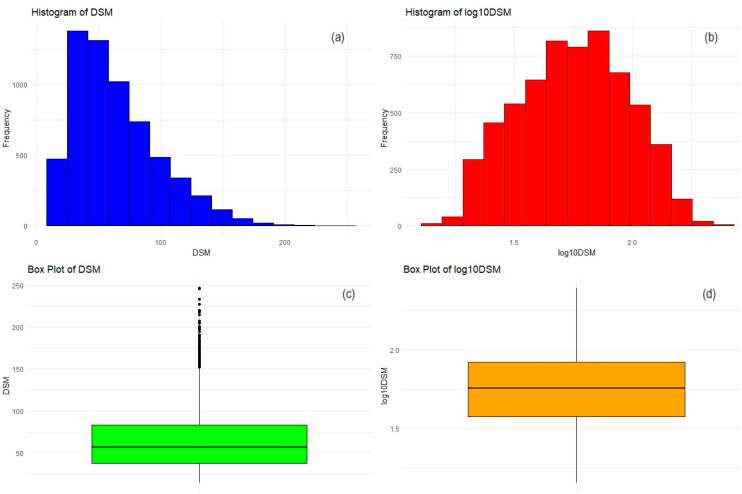
(**a**) Histogram of DSM, (**b**) histogram of log10DSM, (**c**) boxplot of DSM, and (**d**) boxplot of log_10_DSM.

The normality hypothesis was rejected by the Kolmogorov–Smirnov (K-S) test [54,55]. The hypothesis that the series originate from the same distribution was also rejected by the Kruskal–Wallis (K-W) test [56]. The set obtained considering all the series together, denoted by DSM, presented a highly positive skew and outliers (Figure 2), which can pose significant challenges in spatial modeling—Section 2.2.3.

A log10 transformation effectively reduces skewness and diminishes the outliers’ impact by compressing the data’s range, which can be concluded from the K–S results presenting a D statistic score of 0.028086 (suggesting a closer fit to the normal distribution). However, even after this transformation, the dataset did not fully conform to the normality assumptions, as indicated by the small *p*-value of 0.0001204. Figure 2 depicts the histograms and boxplots of DSM before and after the log10 transformation.

### 2.2. Methodology

#### 2.2.1. IDW Interpolation

IDW is one of the oldest spatial interpolation methods used in different research fields [57,58,59], with various versions aiming to improve the parameter selection [60,61,62,63]. IDW estimates values at unsampled locations using the data recorded at neighbor locations. The formula used for this purpose is

(2) $$\hat{z}\left(x_{0}\right)=\left(\sum_{i=1}^{n}\frac{{z(x}_{i})}{d_{i}^{\beta }}\right)/\left(\sum_{i=1}^{n}\frac{1}{d_{i}^{\beta }}\right),$$

where:

*n* is the number of sampling points,

$\hat{z}\left(x_{0}\right)$ is the estimated value at the site $x_{0}$,

${z(x}_{i})$ is the value recorded at the site $x_{i}$,

$d_{i}$ is the distance from $x_{0}$ and $x_{i},$

*β* > 1 is a parameter [64].

According to Equation (1), the closer the location is to $x_{0},$ the higher the contribution in computing the estimated value [64]. The default *β* utilized in most applications is 2, called the inverse squared distance (ISD) interpolator [65]. 

The main disadvantage of this method is the arbitrary choice of *β* and low performances for clustered or unevenly distributed points. Such points located at similar distances from the target location will have approximately the same weight, and so have almost the same contribution to the estimated value [66]. 

In optimizing the IDW model, the focus lies on selecting the most effective power parameter *β* using LOOCV. This optimization process involves evaluating a range of power parameters (from 1 to 10) to determine their influence on the model’s accuracy. During each iteration, for a specific *β*, LOOCV is conducted, wherein the model’s predictive performance is assessed by temporarily omitting the entire records from a point at a time from the dataset [67].

Mean Absolute Error (MAE), Root Mean Squared Error (RMSE), and Mean Absolute Percentage Error (MAPE) were used to assess the model’s performance. These metrics provide a comprehensive model evaluation, with RMSE as the primary criterion for selecting the optimal *β*. The power parameter that results in the lowest RMSE is identified as optimal, ensuring precision and reliability in the model’s spatial predictions.

#### 2.2.2. Spline Smoothing

The key idea behind spline interpolation is to fit a function consisting of local polynomials of degree *p* through the data points. These polynomials describe pieces of a line or a surface and can be constrained to pass through all the known data points while maintaining smoothness. Among the types of splines used in spatial interpolation, the most common is the cubic spline. It is defined over small intervals, and its cubic polynomials are stitched together at specific points, called knots. These knots are the known data points where the curve must pass through. Alternatively, knots away from the data points can be fitted using least squares or other methods to produce smoothing splines [68].

The most significant advantage of cubic spline interpolation (CSI) is the smoothness of the curve, capturing the overall data shape. The basic minimum curvature technique makes CSI more convenient for gently varying surfaces. However, we must be aware that errors can be expected in cases of uneven distribution. In such cases, the tension spline interpolation technique can be applied [68,69].

BSS is an extension of CSI for interpolating data points on a two-dimensional regular grid. The interpolated surface is defined by a piecewise polynomial function in two variables, *x* and *y*. In each rectangular cell formed by the data points, the interpolating function is a bicubic polynomial:

(3) $$z=P(x,y)=\sum_{i=0}^{3}\sum_{j=0}^{3}a_{ij}x^{i}y^{j}$$

where $a_{ij}$ are the coefficients of the polynomial.

We utilized the **‘interp()’** function of the **akima** package in R [70] with the parameter linear = FALSE to implement bicubic interpolation. The method offers smooth surface fitting for our spatial dataset. Despite setting **extrap = TRUE** to enable the extrapolation of values beyond the convex hull of our data points, we encountered instances of NA results, particularly at the edge points of the rectangular grid (points 1, 3–9). Note that, in this context, ‘NA’ signifies missing interpolated values for some locations due to the insufficient nearby data points that BSS requires to compute a value. This limitation arises because the interpolation relies on data that fall within the range of observed values, and points at the periphery may lack sufficient neighboring data to guide the extrapolation process. The issue is particularly pronounced during LOOCV when edge points are omitted, leading to significant uncertainty and the potential for less accurate predictions as the algorithm stretches beyond its reliable bounds.

To address this challenge, our R code introduces a solution by creating a boundary buffer: a series of synthetic points added around the grid’s perimeter. This technique extends the convex hull to include these new points, effectively converting extrapolation scenarios into interpolation ones. The synthetic points are placed just beyond the original grid’s extent, and their associated values are derived from the mean of the nearest actual data points, ensuring a smooth gradient that aligns with the known data distribution. By integrating this boundary buffer into our LOOCV process, we facilitate the **‘interp()’** function’s ability to perform interpolation across the entire grid, including the previously problematic edge points. This approach mitigates the occurrence of NA results and enhances the precision of our spatial predictions, leveraging the strengths of bicubic interpolation across the augmented dataset.

#### 2.2.3. Spatio-Temporal Kriging

Kriging represents a comprehensive suite of generalized least-squares regression algorithms for spatial interpolation. Apart from deterministic methodologies, this approach optimizes weights in a linear predictor to achieve the lowest possible average error in interpolation [71]. STK expands upon the foundational principles of traditional kriging, enabling the analysis of data that encompass both spatial and temporal aspects, which can be particularly advantageous. STK aims to proficiently forecast values at unobserved spatio-temporal points, utilizing the intricate patterns of spatial and temporal correlations within the dataset. STK’s strength lies in its ability to provide statistically robust interpolation, even at boundary points. It can make better-informed predictions at the edges of a grid by considering the spatial relationship of points within the dataset. However, its complexity requires a thorough understanding of the underlying statistical models and assumptions, making it more challenging to implement than simpler methods such as IDW or BSS.

At the core of STK lies the concept of Gaussian processes, which can be thought of as a generalization of the normal distribution of functions. It is a probabilistic model assuming that every point in some continuous input space is associated with a normally distributed random variable [72]. Understanding Gaussian processes helps in comprehending how STK makes predictions at new spatio-temporal points by considering both the mean and variability of the data, providing a statistical framework for our analysis [73].

Considering that a sequence of observations is sampled from a process that can be decomposed into a true spatio-temporal process (assumed Gaussian) and an observational error, the process is expressible through spatio-temporal fixed effects attributed to various covariates. The observational error is modeled as a spatio-temporal-dependent random process. In this case, the process can be modeled by

(4) $$Z\left(s_{ij},\;t_{j}\right)=Y\left(s_{ij},\;t_{j}\right)+\varepsilon \left(s_{ij},\;t_{j}\right),\;i=1,\ldots ,\;m_{t_{j}},\;j=1,\ldots ,\;T,$$

where for each $t\in \left\{t_{1},\;\ldots ,t_{T}\right\}$ there are $m_{j}$ observations [66]. 

This formulation effectively separates the observed data into a deterministic component influenced by specific covariates and a stochastic component that accounts for the randomness inherent in the observation process.

The overarching objective is to employ the dataset z (which includes time- and space-specific observations) to build a model of the random field Z [74] aiming to predict values at unobserved spatio-temporal locations or to enable simulations based on its conditional distribution. It assumes that Z maintains stationarity and exhibits spatial isotropy, allowing it to be defined by a mean function and a covariance function. With an aptly chosen covariance function, one can ascertain the covariance matrices essential for the linear predictor. By applying algebraic methods such as those used in established spatial analyses, it becomes feasible to accurately predict the values of the intended random field [75]. In this article, one covariance model was implemented from the **gstat** package, as described in [74]. The separable model assumes that the global spatio-temporal covariance could be expressed as the product of a spatial and a temporal term, with a variogram given by

(5) $$\gamma_{sep}\left(h,\;\tau \right)=sill\times \left(\bar{\gamma_{s}}\left(h\right)+\bar{\gamma_{t}}\left(\tau \right)-\bar{\gamma_{s}}\left(h\right)\bar{\gamma_{t}}\left(\tau \right)\right),$$

where $\bar{\gamma_{s}}\left(h\right)\;$ and $\bar{\gamma_{t}}\left(\tau \right)$ are the standardized spatial and temporal variograms with separate nugget effects and (joint) sill.

For this study, we explored diverse variogram types (exponential, spherical, Gaussian, and Matérn) that best fit the data series and evaluated them in relation to the covariance model under consideration.

A direct implementation of the above procedure was not possible, given the particularities of the series set. Outliers in spatial and temporal datasets can markedly distort the predictive modeling in kriging, leading to skewed semivariograms and, thus, unreliable interpolation results. Data transformation techniques play a pivotal role in counteracting this. Transformations such as the logarithmic, square root, or more adaptive methods such as the Box–Cox transformation re-scale the data, diminishing the influence of outliers. These transformations compress the range of extreme values, reducing their leverage on the analysis. By normalizing the data distribution and minimizing the variance introduced by outliers, these transformations facilitate a more accurate representation of spatial and temporal autocorrelation, which is central to kriging.

Moreover, pre-processing datasets, particularly the removal of spatio-temporal trends to focus on residuals, is an indispensable step that significantly bolsters the interpretability and precision of kriging outcomes. The detrending process separates the core spatial structure and inherent temporal dynamics from the observed variability, thus enabling kriging to finely tune into the intrinsic autocorrelation of the residuals without the confounding influence of broader trends. Working directly with residuals circumvents the complications introduced by non-stationary behavior attributable to deterministic trends, leading to enhanced prediction accuracy and a profound comprehension of the stochastic elements of the data. Therefore, we adopted a modeling approach using generalized mixed models (GMM) and linear mixed effects (LME) models to remove spatio-temporal trends and work with the residuals. 

To address the normality issue that remained after the log10 transformation (Section 2.1), we initially adopted a straightforward approach using linear models by implementing the **lm()** function, which fits linear surfaces to the data. This first step involves exploring simple relationships, such as first-order (straight) or second-order (curved) surfaces, to understand the basic trends in the series set. However, the data series is collected over sea and desert areas, and these basic linear models often fail. They might not adequately capture the complex and nuanced relationships inherent in the data, such as the non-linear patterns and the unique characteristics of different sampling points. Therefore, we transitioned to more advanced statistical models such as generalized additive models (GAM), GAMM, and LME [76].

GAMs are particularly adept at uncovering complex, non-linear relationships within a data series. For instance, they can help us understand how the concentration levels might increase non-uniformly in response to changing environmental factors such as humidity or temperature. This aspect is particularly relevant when considering the diverse nature of the data from various geographical locations. Building on this, GAMM adds another layer of sophistication by incorporating ‘random effects’. These are useful in our scenario, where each of the 70 distinct points presents a unique environmental characteristic, allowing modeling these differences while analyzing the overall data trends.

LME models come into play due to their strength in handling linear relationships and their capability to address fixed effects (common trends across all points) and random effects (unique characteristics of each point). In the context of our dataset, which exhibits a clear seasonal pattern with peaks every six months, LMEs are particularly suitable. They can effectively capture these predictable temporal trends while accounting for spatial variations across locations. By focusing on the residuals from the LME component, we can discern aspects of the seasonal variation and the site-specific differences that simpler linear models do not fully explain.

**Figure 3:**
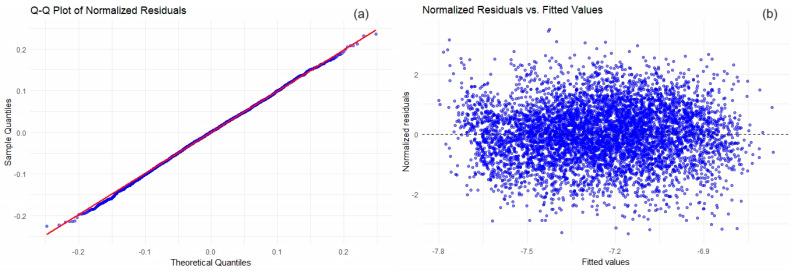
Diagnostic plots for GAM model residuals analysis. (**a**) Q-Q plot of the normalized residuals; (**b**) normalized residuals vs. fitted values.

GAMM from the **‘mgcv’** package in R was utilized to model the spatial field and temporal trends. The thin plate regression splines’ **t2()**’ within GAMM allowed for flexible shaping of the spatial and temporal effects, capturing the complex underlying patterns in the data. The ‘**gamm()**’ function was used to fit these models, which internally uses the ‘**lme()**’ function to estimate random effects, providing a robust framework for detrending the dataset. After fitting the GAMM, we extracted the residuals representing the detrended data—the pure spatial-temporal stochastic component we aim to model with kriging. The residuals follow a normal distribution almost perfectly with an adequate, stable variance as indicated by a D = 0.013273 and a *p*-value = 0.2279 of the K–S test and by the visual verification of diagnostic plots in Figure 3. 

For LOOCV, we systematically removed each station from the dataset and used the remaining stations to predict the left-out station’s values. We performed spatio-temporal kriging on these residuals, ensuring that the predictions were based on the stochastic properties rather than any underlying deterministic trends.

The final step involved back-transforming the predictions to the original series scale to interpret the results in their natural units. It was accomplished by first predicting the trend component for each data point using the ‘**predict()**’ function on the fitted GAMM model. Then, we combined this trend component with the kriged residuals to obtain the final predictions in the log10 scale. To correct the bias introduced by the log transformation, a correction factor of $10^{\sigma^{2}/2}$ was calculated, where $\sigma^{2}$ is the variance of the log10-transformed predictions. The final predictions were then scaled back to the original series units by reversing the log10 transformation and applying the correction factor. This back-transformation is crucial as it allows the results to be presented on the same scale as the original measurements, making them directly comparable and interpretable in the context of the study.

The entire LOOCV process was automated in R software, where we evaluated various variogram models, including spherical, exponential, Gaussian, and Matérn, to accurately capture the dataset’s complex spatio-temporal dynamics. This systematic approach involved fitting each model to the detrended data to identify the ones that best represent the spatio-temporal correlations. We kept the spatio-temporal combination of variogram models that yielded the lowest MSE. 

To gauge the overall performance of the models, MAE, RMSE, and MAPE were calculated for each station and then averaged across all periods. MAE provides the evaluation of the systematic error. RMSE better explains the random errors. RMSE is valuable in situations where significant errors are particularly undesirable, as it helps identify models with occasional large deviations from the actual values. MAPE is beneficial when dealing with data where the magnitude varies significantly or when comparing the accuracy of models across different data scales. Values close to zero indicate the models’ good performances. 

Three more efficiency indicators were also used for comparing the models:

- Nash–Sutcliffe Efficiency (NSE) is a normalized statistic that determines the relative magnitude of the residual variance compared to the measured data variance. It is defined by
  (6) $$NSE=1-\frac{\sum_{i=1}^{N}{\left(Q_{obs,i}-Q_{sim,i}\right)}^{2}}{\sum_{i=1}^{N}{\left(Q_{obs,i}-{\bar{Q}}_{obs}\right)}^{2}}$$
  where $Q_{obs,i}$ = observed data, $Q_{sim,i}$ = simulated data, ${\bar{Q}}_{obs}$ = mean of observed data, and *N* = number of time steps.
  The range of NSE is (−∞, 1). NSE = 1 indicates a perfect match between the model and the observations. A value of 0 shows that the model predictions are as accurate as the average of the observed data. Still, NSE is sensitive to both high and low values.
- Kling–Gupta Efficiency (KGE) decomposes the NSE into components representing correlation, variability, and bias, providing a more comprehensive measure of model performance. The formula is
  (7) $$KGE=1-{\left(r-1\right)}^{2}+{\left(\alpha -1\right)}^{2}+{\left(\beta -1\right)}^{2}$$
  where *r* = correlation between observed and simulated data, *α* = ratio of the standard deviation of simulated to observed data, and *β* = ratio of the mean of simulated to observed data.
  The benefits of using KGE are as follows:
  (1) The decomposition allows us to diagnose which aspect of the model is contributing to inefficiencies.
  (2) KGE balances different performance aspects, providing a more holistic view of model performance.
  (3) Using it with NSE to assess model performance leads to making informed decisions about model improvements.
- Index of Agreement (dIndex) is a standardized measure of model prediction error and represents the ratio of the mean square error and the potential error. It is given by
  (8) $$dIndex=1-\frac{\sum_{i=1}^{N}{\left(Q_{obs,i}-Q_{sim,i}\right)}^{2}}{\sum_{i=1}^{N}{\left(\left|Q_{sim,i}-{\bar{Q}}_{obs}\right|+\left|Q_{obs,i}-{\bar{Q}}_{obs}\right|\;\right)}^{2}}.$$

DIndex measures the magnitude of the error and its pattern and distribution. It is effectively an agreement measure, assessing how well the predicted data match the observed data range. Moreover, it can be used across different disciplines, including hydrology, climatology, and ecology.

**Figure 4:**
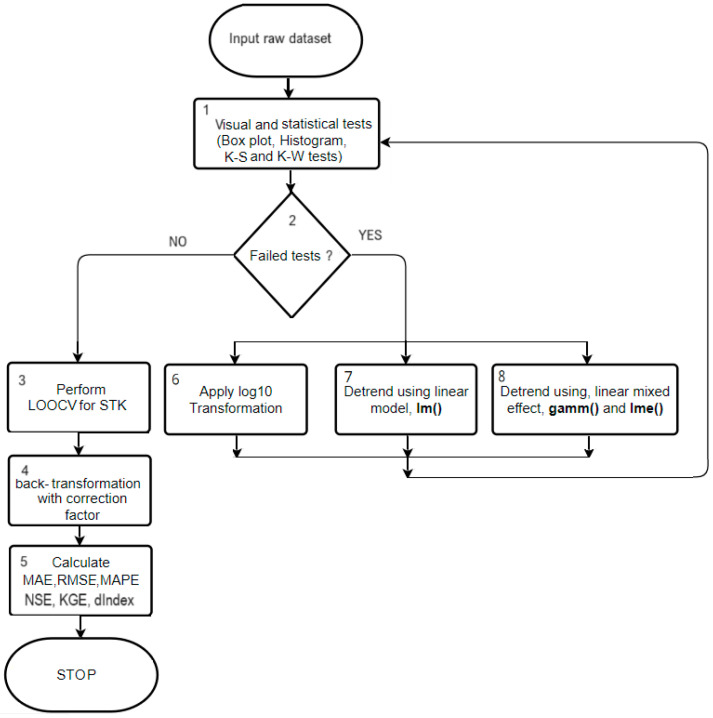
Workflow diagram for STK analysis of data series.

Finally, we present a comprehensive workflow diagram (Figure 4) and its corresponding algorithmic description. Algorithm 1 details the step-by-step process of analyzing data series through STK, including data preprocessing, transformation, and validation stages, culminating in the prediction and error quantification phases.

**Algorithm 1.** The algorithm for the data series interpolation using STK has the following stages
0.Input: Raw dataset1.Perform Exploratory Data Analysis (EDA) on raw datasetVisual inspections: Box plot, HistogramPerforms the K-S and K-W tests2.Assess EDA Results: IF the dataset passes normality and homoscedasticity tests, THEN GOTO Step 3, ELSE, GOTO Step 6.3.Perform LOOCV for STK4.Back-transformation of Predicted Data:Predict the trend for each data point (if data was detrended in Steps 7 or 8)Apply the correction factor and reverse any log transformations5.Calculate Error Metrics: MAE, RMSE, and MAPEEND Algorithm6.Data Transformation (IF needed after Step 2)Apply log10 transformation to the dataGOTO Step 1 (Reapply EDA)7.Simple Linear Detrending (IF Step 6 fails to normalize data)Detrend data using a simple linear model with the ‘lm()’ functionGOTO Step 1 (Reapply EDA)8.Complex Model Detrending (IF Step 7 fails to normalize data)Detrend data using complex models like GAMM and LMEGOTO Step 1 (Reapply EDA)


## 3. Results and Discussion

In the presentation of the results, we strategically select points from the spatial grid, chosen to represent diverse locations: points 1, 10, 61, and 70 representing the corners, point 35 representing the central area and surrounded by a higher density of data points, and points 5, 30, 31, 65 representing an edge of the grid, allowing us to demonstrate the robustness of each model under various spatial scenarios, ranging from data-rich central areas to potentially data-sparse edges and corners. Furthermore, we globally assess errors across all stations for each method. The key error metrics—MAE, RMSE, and MAPE—are computed for these predictions.

### 3.1. IDW Results 

**Figure 5:**
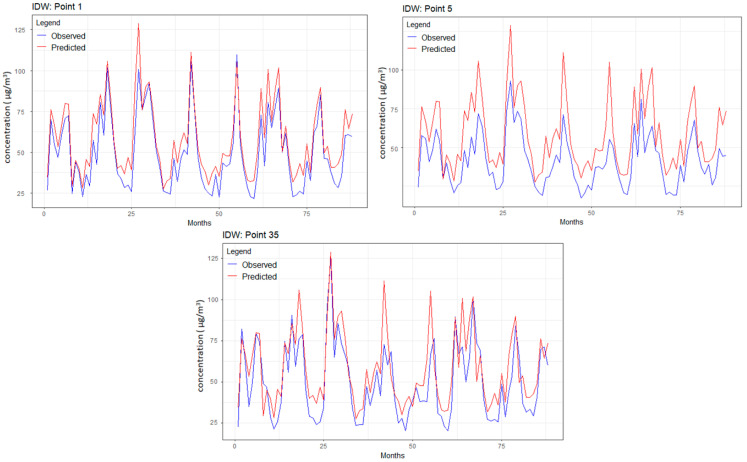
Observed and predicted values of the series from points 1, 5, and 35 (the months are counted from 1 (January 2010) to 88 (April 2017).

Figure 5 depicts the predicted and observed series values for the study period for points 1, 5, and 35. In all cases, the forecast values were higher than the data series, but the predicted series had the same shape as the raw one. The goodness of fit parameters for the nine points above are presented in Table 2, together with the minimum, maximum, and average MAEs, RMSEs, and MAPEs computed for all the sampling points.

MAEs and RMSEs were extremely low, indicating the goodness of fit of IDW with the optimum parameters *β*. The highest MAPE among the points mentioned above corresponded to point 1, located above the Gulf in the northeastern corner of the grid due to uneven distribution of the neighbors. By comparison, MAPEs computed for the points in the other corners (3.115, 3.848, and 5.524) were under the average MAPE, indicating a good fit for the data series. 

The series from the points in the central areas were not the best fit given the contribution of the series inside the UAE and over the Arabian Gulf (with different concentrations of PM_2.5_). The first incorporates the PM_2.5_ originated from industrial sources and dust storms from the desert (particularly in spring and the beginning of autumn) [10,11]. The second reflects the influence of the dust transported over the Arabian Gulf from North Africa [77,78,79,80].

This approach to optimizing the IDW model underscores a balance between methodological rigor in parameter selection and flexibility in model customization, ensuring robustness and adaptability in spatial data analysis. 

### 3.2. Results of BSS

**Figure 6:**
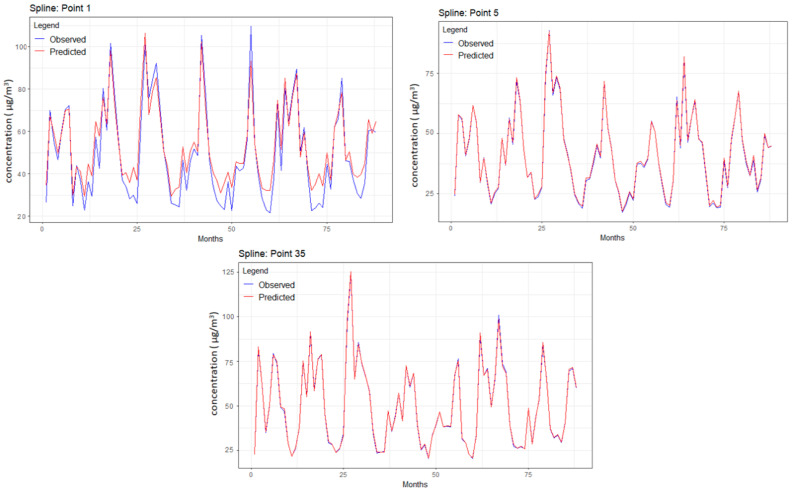
Observed and predicted concentration series for points 1, 5, and 35.

All predicted series showed an excellent concordance with the recorded series, as exemplified in Figure 6 for points 1, 5, and 35. The worst performances were recorded in point 1 (at the grid corner) compared to 5 and 35.

The explanation is that the corners or sides have a smaller number of neighbors, so a lower estimation quality is expected. Among the points in the grid corners, the series from 1 was also the worst fit, whereas the best fit was the series registered at point 61. Remember that the original spline algorithm returned NA for the series from 1, and the buffering technique implemented here might introduce some errors in the series forecast. Even in these conditions, the MAE, RMSE, and MAPE corresponding to point 1 (Table 3 column 5) were not the highest among the other MAPEs, indicating that the proposed approach yielded good results in forecasting the PM_2.5_ series. The same is true for the series from points 3–9, for which the algorithm provided outstanding results in terms of all goodness-of-fit indicators (with values lower than the corresponding averages). 

Table 3 presents the MAE, RMSE, and MAPE for the same selected points, and the maximum, minimum, and mean values of the goodness of fit parameters for all 70 points. MAPE for point 61 was close to the minimum MAPE. Moreover, MAEs and RMSEs for all the series recorded at the points on the grid edges were under the average MAE and RMSE, indicating the fit quality provided by the implemented interpolation procedure.

Other approaches can be explored to refine the BSS potential. One could use a weighted average that considers the distance of each neighboring point, giving closer points a greater influence on the value of the synthetic point.

Another might involve spatial trend analysis, where the synthetic points’ values are informed by identified spatial gradients or patterns within the data—this could be particularly useful if the variables show systematic changes across the study area.

### 3.3. Results of STK

Table 4 contains the minimum, maximum and average values for the selected points analyzed in the previous sections. Among the series displayed in Table 4, the best fit was the series from point 35 (situated inside the grid), followed, unexpectedly, by that from the grid corner—point 10. 

**Figure 7:**
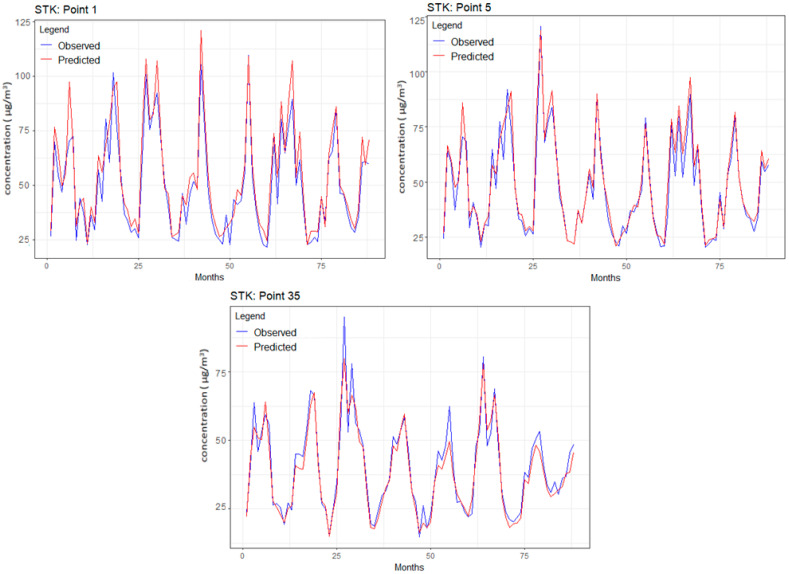
Observed and predicted series for points 1, 5, and 35.

Figure 7 depicts the predicted and observed values for the series from points 1, 5, and 35, respectively. A visual investigation of the chart from Figure 7 indicates that the predicted series mostly slightly overestimated the recorded ones. The series extremes are not always captured very well. These remarks are confirmed by the MAPE, which varied between 7.656 and 20.863. 

Considering the transformation performed on the data series, it was expected to obtain slightly higher errors than in the other models since each operation introduces additional errors.

### 3.4. Discussion

In this study, we compared three distinct interpolation methods, IDW, an adapted Spline method, and STK, to interpolate the PM_2.5_ concentration series. Comparing the MAE, MSE, and MAPE results in the following:

- The lowest minimum and maximum MAE, RMSE were obtained using IDW.
- The lowest minimum (maximum) MAPE was obtained utilizing IDW (STK).
- The lowest average MAE, MSE, and MAPE resulted from BSS.
- BSS best fitted the series from the grid corners 1, 61, and 70, whereas the values of series from 10 were better fit by IDW.
- The series on the edges were mostly best fit (at least in terms of MAPE) by BSS.
- The most difficult to forecast were the series on the grid corners and edges due the neighbors’ positions.

Using the non-parametric Friedman test [81], which is particularly suited for datasets without a normal distribution, we tested the hypothesis that there is no difference between the performance of these methods (based on MAE, RMSE, and MAPE) against the existence of some differences. The test results showed significant disparities (*p*-values < 2.2 × 10^−16^ for MAE and RMSE, and *p*-value = 1.085 × 10^−15^ for MAPE). A Nemenyi post-hoc test [82] further clarified these findings, indicating that our adapted spline method outperformed IDW and STK in all error metrics. Specifically, it exhibited a reduction in mean errors by approximately 21% in MAE, 15% in RMSE, and 21% in MAPE compared to IDW, and around 60% in MAE, 61% in RMSE, and 58% in MAPE compared to STK. These high-performance results of the spline method can be attributed to the specific adaptations for handling boundary and corner points, underscoring its effectiveness and precision in interpolating the PM_2.5_ concentrations.

**Figure 8:**
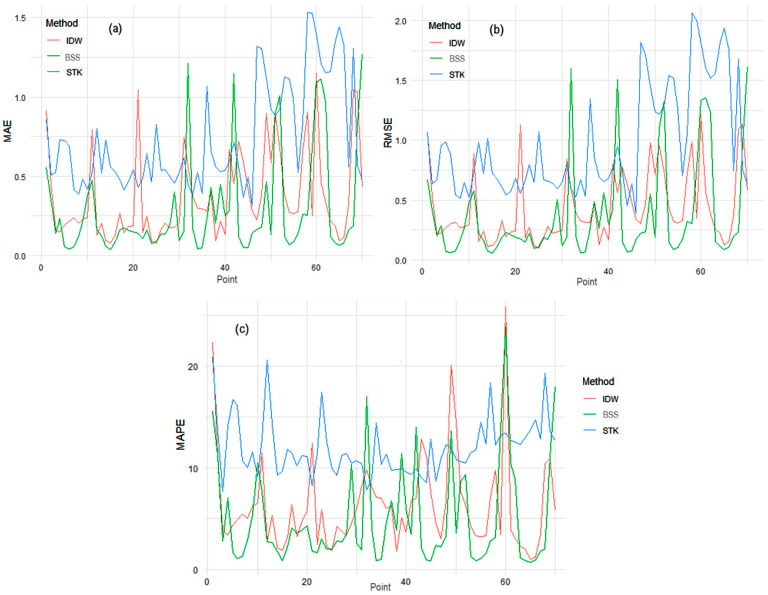
(**a**) MAE, (**b**) RMSE, and (**c**) MAPE variation by method across stations.

Figure 8 depicts the variation in the error metrics across all the 70 points. 

When working with IDW, the distance from the target point to the neighbor and the degree of similarity between the data series are essential. The positive spatial autocorrelation affects the IDW’s performance [83] because the similarity of the series collected in neighboring locations is more probable than that of the series situated at a higher distance from each other [84]. These findings are consistent with those from [6], which indicated that the IDW effectiveness is notable in regular grid distributions, like in this study case. Limitations were noticed at grid edges, where the absence of neighbors can alter the interpolations. This boundary effect is also a challenge in BSS.

BSS can also fail at the edge or corners of the grid (like in the initial attempt of our study) because the smoothness and continuity hypotheses on which they rely may not be satisfied at the mentioned points due to the limited number of data [58,85]. This is an important issue when the models are used for forecasting pollutant series, such as in the present case. In this study, we overcame this limitation by creating a boundary buffer that led to the best forecast among the three considered methods.

Although STK can potentially improve prediction accuracy by integrating data across time and allowing the assessments at specific points or across entire fields during and beyond observation periods, this method must account for complex space–time variability, which is often more challenging than spatial or temporal methods alone. Dependence structures, such as variograms, vary and may require an anisotropy parameter for simplification, introducing significant assumptions about the observed processes [75]. Furthermore, the covariance function separability assumption is rarely valid for real-world processes. It implies that the temporal pattern observed at a specific location is independent and not directly influenced by the temporal patterns at other locations [71]. For this reason, one should apply more complex covariogram function formulations such as sums and products. 

**Figure 9:**
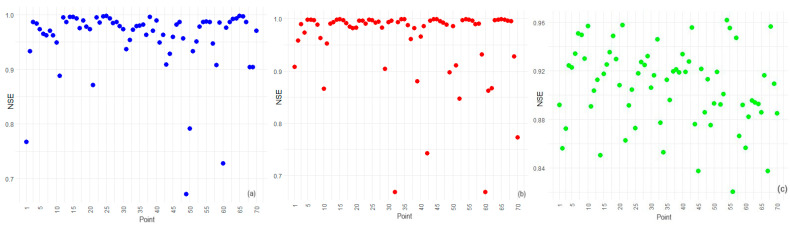
NSE for (**a**) IDW, (**b**) BSS, and (**c**) STK.

**Figure 10:**
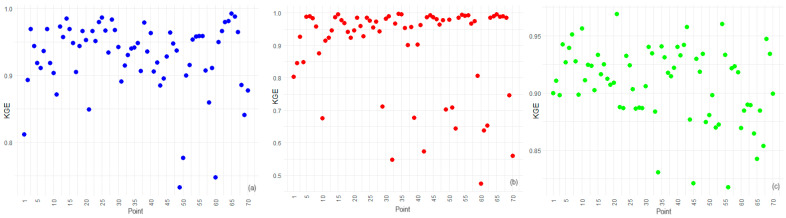
KGE for (**a**) IDW, (**b**) BSS, and (**c**) STK.

**Figure 11:**
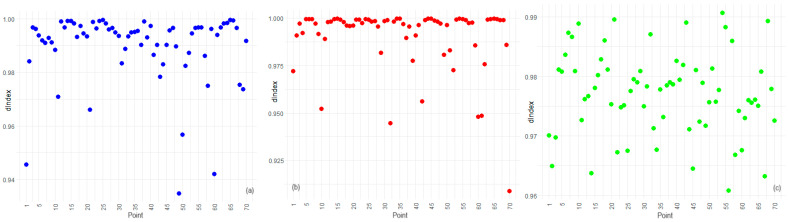
dIndex for (**a**) IDW, (**b**) BSS, and (**c**) STK.

Figure 9, Figure 10 and Figure 11 contain the plots of the NSE, KGE, and dIndex for IDW, BSS, and STK. In our spatial analysis, the station-specific performance of interpolation methods is evident from the NSE, KGE, and dIndex.

Edge and corner stations, such as points 1 and 70 at the periphery and points 10 and 11 at coastal boundaries, are particularly informative in understanding the spatial limitations of these methods.

The BSS method’s high NSE and dIndex values across the board, including edge stations such as point 1 and coastal corners such as point 10, demonstrate its robustness and spatial consistency. This result aligns with findings from the Friedman and Nemenyi tests, which identified BSS as the superior method in terms of overall error metrics. The method’s strength is not compromised by the spatial position of the stations, indicating its suitability for varied terrain and its resilience to edge effects.

For IDW, lower KGE values at points on the periphery suggest that the spatial distribution of points somewhat influences its performance. Since IDW relies on the proximity and weighting of nearby points, stations with fewer neighbors, such as point 70, may experience reduced accuracy, particularly in capturing variability and bias. These findings highlight the method’s spatial sensitivity, necessitating careful application or potential methodological adjustments in areas where data points are sparse or unevenly distributed.

STK’s KGE and dIndex results, particularly at edge stations such as point 21 and corner locations such as point 11, imply that this method’s assumptions may not uniformly accommodate the unique spatial-temporal dynamics present at these stations. The broader spread in efficiency scores for STK across different stations underscores the method’s nuanced application, warranting a closer look at the spatial-temporal structuring of the data in these areas.

Moran’s I [86] and Geary’s C [87] tests, which showed no significant spatial autocorrelation in residuals, reinforce the interpretation that the observed variations in efficiency and agreement are not due to spatial patterning in the data but rather reflect the interaction between the methods’ inherent characteristics and the environmental context of each station.

While BSS demonstrates versatility and reliability across the spatial domain, IDW and STK may require station-specific considerations, particularly at spatially constrained edge and corner locations. Kriging or other geostatistical methods could be applied to estimate the values of the boundary points by modeling the spatial autocorrelation of the data. This alternative could provide another way to create the boundary buffer, potentially offering improvements in the interpolation accuracy and the robustness of the overall spatial model. This holistic assessment, integrating both statistical performance and spatial considerations, ensures a well-informed approach to PM_2.5_ variable modeling, addressing the intricacies of the spatial and temporal variability inherent in PM_2.5_ data. 

The methods proposed here can be applied for spatial interpolation of any spatially distributed data series. 

The literature search returned studies providing mostly IDW applications in different fields and comparisons with kriging and other methods but not STK and BSS. Li and Heap [58] compared 72 methods provided by 53 articles. They found that IDW, ordinary kriging (OK), and ordinary co-kriging (OCK) are the most utilized approaches. IDW outperformed some methods in a few cases [88,89], has similar performance [90,91], or is worse than OK in other cases [63,92]. For example, IDW, the principal component regression with the residual correction (PCRR), and the multiple linear regression (MLR) were employed to compare interpolated precipitation at different scales and the spatial distribution of rainfall in a catchment in China [93]. The results showed that the PCRR method was the best. IDW, OK, OCK Nearest Neighbor (NN), Trend Surfaces and Regression (TSR), TSA-OK, and Bayesian Model Averaging (BMA) were compared in the study [94], showing that TSA-OK and BMA performed best. 

In its study, Dubrule [95] indicated that splines and kriging should be used alternately, depending on the purposes, but from the accuracy viewpoint, kriging is better. A comparison of spatio-temporal interpolation of geographic data using reduction and extension methods with spatio-temporal interpolation methods based on IDW and OK indicated the worst results among the last two methods [96]. 

Different approaches have been used for the spatio-temporal interpolation, but only a few applied BSS and STK, without providing comparisons [97,98,99,100,101,102,103,104,105]. The least studied is BSS interpolation [85,106]. Therefore, deeper investigations must be carried out on various data series to assess the performances of BSS and STK.

## 4. Conclusions

This study concludes that the BSS model stands out among the three models evaluated for the PM_2.5_ concentration in the Arabian Gulf due to its unique approach to boundary conditions. As a first contribution novelty of the study, implementing buffer points significantly reduces error metrics, establishing the BSS method’s superiority in accuracy over the IDW and STK methods. Therefore, it is recommended for forecasting the PM_2.5_ in locations where records are unavailable. 

While the IDW model, in its basic form with an optimized power parameter *β* for the complete temporal records, is promising, it could benefit from enhancements in boundary effect handling. Such improvements could potentially elevate its accuracy, especially at grid edges. This approach could involve integrating strategies similar to those used in the BSS model, such as introducing buffer points or advanced weighting algorithms near boundaries. 

STK, known for its robust integration of spatial and temporal data, suggests potential for refinement in future research. Exploring different covariogram functions and optimizing variogram parameters could lead to a more accurate representation of the complex spatial-temporal characteristics of the PM_2.5_ series. Such advancements would not only enhance the model’s accuracy but also its applicability in environmental monitoring. These findings underscore the efficacy of each method in spatio-temporal interpolation and the potential for further optimization, contributing to our understanding of PM_2.5_ distribution and serving as a model for other regions facing similar environmental challenges. 

Apart from the originality of using a GAMM-STK approach for non-normally distributed series and proposing the buffering for BSS, after a deep literature research, this study was evidenced as unique by comparing the proposed approaches on spatial-temporally distributed data series. Moreover, the methods can be used for any data series and region. Future research will explore other optimizations and their implications for spatio-temporal data analysis in various environmental contexts. 

The article fills in a gap related to the PM_2.5_ distribution in the UAE, providing results that can be the background for studies on the effects of pollution on the population. These results can also be used by policymakers interested in reducing PM_2.5_ pollution in the UAE.

## Figures and Tables

**Table 1:** Extreme values of the PM_2.5_ data series’ basic statistics.

	Min (μg/m^3^)	Max (μg/m^3^)	Average (μg/m^3^)	Stdev (μg/m^3^)	Cv (%)	Skew	Kurt
min	14.30	80.10	36.49	13.19	29.69	0.07	−1.10
max	51.90	246.00	119.36	37.34	46.15	1.17	1.92

**Table 2:** MAE, RMSE, and MAPE in the IDW interpolation with the optimal *β*.

	**min**	**max**	**average**	**Point 1**	**Point 5**	**Point 35**
MAE	0.26	10.5	3.33	8.62	1.85	2.88
RMSE	0.037	11.2	4.03	10.3	2.67	3.13
MAPE	0.544	22.8	5.58	21.478	4.161	6.957
	**Point 10**	**Point 61**	**Point 70**	**Point 30**	**Point 31**	**Point 65**
MAE	1.26	4.58	4.16	2.05	7.3	0.7
RMSE	2.19	5.59	5.74	2.46	8.37	1.09
MAPE	3.115	3.848	5.524	5.863	7.955	0.737

**Table 3:** MAE, RMSE, and MSE in the splines interpolation after using buffering points.

	**min**	**max**	**average**	**Point 1**	**Point 5**	**Point 35**
MAE	0.38	12.69	2.96	5.55	1.22	2.51
RMSE	0.55	16.11	3.71	6.72	1.64	2.96
MAPE	0.709	23.834	5.022	15.556	2.623	5.961
	**Point 10**	**Point 61**	**Point 70**	**Point 30**	**Point 31**	**Point 65**
MAE	1.77	0.81	2.16	2.29	4.29	1.90
RMSE	2.30	1.14	2.69	2.61	4.86	2.31
MAPE	3.569	0.844	5.363	4.671	6.772	2.002

**Table 4:** MAE, RMSE, and MSE in the STK interpolation.

	**min**	**max**	**average**	**Point 1**	**Point 5**	**Point 35**
MAE	3.14	15.31	7.37	8.58	7.24	3.92
RMSE	3.86	20.62	9.64	10.65	9.85	5.29
MAPE	7.656	20.863	12.000	20.863	16.717	10.319
	**Point 10**	**Point 61**	**Point 70**	**Point 30**	**Point 31**	**Point 65**
MAE	4.17	12.13	5.35	5.23	6.20	14.40
RMSE	5.19	16.06	7.02	6.48	7.93	19.34
MAPE	9.107	14.183	12.693	10.672	10.425	13.677

## Data Availability

Data are freely available at: https://disc.gsfc.nasa.gov/datasets/M2T1 NXLND_5.12.4/summary (accessed on 10 May 2022).

## Appendix A

**Table A1:** Coordinates of the grid points.

ID	X	Y	ID	X	Y	ID	X	Y	ID	X	Y	ID	X	Y
1	51.2500	26.3125	15	53.7500	25.6875	29	56.2500	25.0625	43	52.5000	23.8125	57	55.0000	23.1875
2	51.8750	26.3125	16	54.3750	25.6875	30	56.8750	25.0625	44	53.1250	23.8125	58	55.6250	23.1875
3	52.5000	26.3125	17	55.0000	25.6875	31	51.2500	24.4375	45	53.7500	23.8125	59	56.2500	23.1875
4	53.1250	26.3125	18	55.6250	25.6875	32	51.8750	24.4375	46	54.3750	23.8125	60	56.8750	23.1875
5	53.7500	26.3125	19	56.2500	25.6875	33	52.5000	24.4375	47	55.0000	23.8125	61	51.2500	22.5625
6	54.3750	26.3125	20	56.8750	25.6875	34	53.1250	24.4375	48	55.6250	23.8125	62	51.8750	22.5625
7	55.0000	26.3125	21	51.2500	25.0625	35	53.7500	24.4375	49	56.2500	23.8125	63	52.5000	22.5625
8	55.6250	26.3125	22	51.8750	25.0625	36	54.3750	24.4375	50	56.8750	23.8125	64	53.1250	22.5625
9	56.2500	26.3125	23	52.5000	25.0625	37	55.0000	24.4375	51	51.2500	23.1875	65	53.7500	22.5625
10	56.8750	26.3125	24	53.1250	25.0625	38	55.6250	24.4375	52	51.8750	23.1875	66	54.3750	22.5625
11	51.2500	25.6875	25	53.7500	25.0625	39	56.2500	24.4375	53	52.5000	23.1875	67	55.0000	22.5625
12	51.8750	25.6875	26	54.3750	25.0625	40	56.8750	24.4375	54	53.1250	23.1875	68	55.6250	22.5625
13	52.5000	25.6875	27	55.0000	25.0625	41	51.2500	23.8125	55	53.7500	23.1875	69	56.2500	22.5625
14	53.1250	25.6875	28	55.6250	25.0625	42	51.8750	23.8125	56	54.3750	23.1875	70	56.8750	22.5625
